# Functionality appreciation is associated with improvements in positive and negative body image in patients with an eating disorder and following recovery

**DOI:** 10.1186/s40337-023-00903-y

**Published:** 2023-10-09

**Authors:** Manja M. Engel, E. M. Woertman, H. C. Dijkerman, A. Keizer

**Affiliations:** 1 Department of Experimental Psychology, Faculty of Social and Behavioural Sciences, Utrecht University, 3584 CS Utrecht, The Netherlands; 2 Department of Clinical Psychology, Faculty of Social and Behavioural Sciences, Utrecht University, Utrecht, The Netherlands

**Keywords:** Positive body image, Negative body image, Functionality appreciation, Eating disorders, Recovered eating disorder patients

## Abstract

**Background:**

Research on body image in eating disorders has predominantly focused on negative body image, only recently shifting to positive body image. Findings suggest that enhancing positive body image can, amongst other things, serve as a protective mechanism against (re)developing a negative body image. One suggested way of enhancing positive body image is to focus on enhancing body functionality appreciation. Although studies show promising effects, this research is mainly conducted in non-clinical samples.

**Methods:**

The current study investigated the levels of positive and negative body image in an online community sample of patients with an eating disorder (PAT, *n* = 227), patients recovered from an eating disorder (REC, *n* = 102) and controls (HC, *n* = 175) (self-reported diagnosis, not confirmed). In addition, we tested whether body functionality appreciation was associated with appearance dissatisfaction (negative body image) and body appreciation (positive body image).

**Results:**

REC showed similar results to controls to most of the body image measures except for how much importance one places on their appearance (no different between REC and PAT), and how satisfied one is with certain body parts. For this measure, REC scored in-between PAT and HC. We further found functionality appreciation to be significantly associated with of both positive and negative body image, except for appearance evaluation in patients with an eating disorder.

**Conclusions:**

This study showed a positive association between body functionality appreciation and positive body image and a negative association between body functionality appreciation and negative body image. Further research is required to investigate the effectiveness of interventions targeting body functionality appreciation in clinical settings.

## Abstract (plain language summary)

People with an eating disorder often suffer from a negative body image. This negative body image has been found to be hard to treat. Recently researchers started to not only look at ways to decrease negative body image but also enhance positive body image. It has been found that it is beneficial for people with a negative body image to learn to focus on appreciating the functions of the body (body functionality appreciation), as this leads to a more positive body image. However, this research was done on people without an eating disorder. In this questionnaire study, we looked at whether there is a relationship between body functionality appreciation and body image in patients with a (self-reported) eating disorder diagnosis and those who are recovered from an eating disorder. We found that functionality appreciation is associated with increased positive body image and decreased negative body image in all groups. Further studies should investigate the effectiveness of clinical interventions that focus on body functionality appreciation to enhance body image

## Introduction

People with an eating disorder often suffer from an unjustified feeling of being fat. This phenomenon is part of the broader concept of body image disturbance. Body image has seen many definitions, but it is mostly referred to as a multidimensional construct that includes body attitudes (beliefs and emotions) and perception [1]. A disturbance in body image is associated with various psychiatric illnesses and is frequently seen in eating disorders. Body image disturbance is even a diagnostic symptom for anorexia nervosa, bulimia nervosa and other specified feeding or eating disorder [2]. It is also frequently found in binge eating disorder [3].

The importance of body image disturbance in eating disorders is reflected in various findings. For example, body image disturbance is positively associated with the onset and maintenance of eating disorders [4–7], and a predictor of relapse [8]. Furthermore, body image disturbance is a complicating factor for recovery; higher baseline preoccupation with shape and weight (body attitudes) at the start of inpatient treatment is associated with slower rates of improvement in eating concern, general psychopathology, and work and social functioning [9]. Thus, research focusing on body image disturbance is important to optimize treatment in eating disorders.

Research on body image disturbance in eating disorders has traditionally focussed on *negative* body image [10], where the focus lies on negative body attitudes. These include discontent with aspects of one’s physical appearance (body dissatisfaction) and preoccupation with and overevaluation of body size [11]. It has been found that patients with an eating disorder are generally more dissatisfied with their appearance and are overly concerned with their body size and shape compared to controls [12]. For example, research has shown that patients have a more negative appearance orientation, evaluate their appearance more negatively, have a higher overweight preoccupation, and a lower satisfaction with specific body areas compared to controls [13, 14].

In the past decade some researchers have focussed on *positive* rather than negative body image. While the concept of negative body image emphasises physical appearance and weight [10], positive body image has a broader definition. Tylka and Wood-Barcalow [15] defined positive body image as a multifaceted construct (including body appreciation, body acceptance/love, conceptualizing beauty broadly, adaptive investment in appearance, inner positivity, interpreting information in a body-protective manner) that has a holistic character, it and is linked to self-perceived body acceptance by others, and shaped by social identities. Furthermore, it was found that a specific aspect of positive body image, body appreciation, decreases eating pathology, and protects against eating pathology symptoms in patients with an eating disorder [16].

Positive body image is distinct from negative body image. Tylka and Wood-Barcalow [15] argue that body image should not be conceptualized on a continuum, with negative and positive body image at both endpoints. There are indeed several studies in non-clinical populations showing that positive body image and negative body image are two unique constructs [15]. Such studies have shown that people are able to experience some level of body appreciation and body dissatisfaction simultaneously [17]. For example, someone can be happy with their appearance but still desire to change the shape of their abdomen [18].

If positive and negative body image are indeed two separate constructs, it could be that interventions targeting negative body image, might only reduce symptoms of negative body image, instead of helping individuals to adopt a positive body image. In other words, treating negative body image may not automatically lead to an improvement in positive body image. In the most positive scenario, interventions on negative body image would lead to a neutral body image where individuals would merely tolerate their bodies [15]. Merely tolerating one’s body might not be sufficient for treating patients with an eating disorder as patients are more prone to (re)develop a negative body image compared to individuals without an eating disorder [19]. As negative body image is a predictor for relapse [8], treatment should not only focus on reducing negative body image but should also focus on enhancing *positive* body image [15, 16, 20, 21]. Enhancing positive body image would mean that patients would not only develop a positive body image (instead of neutral, tolerating, body image), but they would also be protected against (re)developing a negative body image.

One suggested way to enhance positive body image is to focus on body functionality appreciation, which involves ‘appreciation of what the body is capable of doing’ [20]. Appreciation of the body’s function is in line with the broader concept of the positive body image, where the idea is to focus not on what the body looks like, but on appreciation for what it can do. Body functionality appreciation is defined as appreciating, respecting, and honouring of what the body is capable of doing. The bodily functions include physical capacities, but also internal processes, senses and sensations of the body, and verbal and non-verbal communication [20–22]. While body functionality appreciation is considered a component of positive body image, it is nevertheless distinct from other components such as body appreciation and body acceptance [21].

Focusing on what the body is capable of doing, as opposed to what the body looks like, has shown to improve body image, specifically, appearance satisfaction, functionality satisfaction, body appreciation, and lower self-objectification, in a non-clinical sample of women with negative body image [20]. In other words, higher levels of functionality appreciation both decreases negative body image (dissatisfaction with appearance) and increases positive body image (appreciation of the body). Given these findings, different researchers have suggested that focussing on body functionality appreciation has a beneficial effect in reducing overemphasis on and overvaluation of physical appearance [for an overview see: 22].

While studies on the positive effect of body functionality on body image have been predominately conducted in non-clinical samples, Rekkers and Boerhout [23] suggested that helping patients with an eating disorder shift their focus to body functionality is a useful technique for improving body image in clinical treatment for eating disorders [25]. However, body image in terms of functionality appreciation is seldom targeted in standard treatment, which predominantly focuses on patients’ problems regarding appearance [24]. Even though functionality appreciation has been effective in improving both positive and negative body image in a non-clinical sample [20], to our knowledge only one (pilot) study has shown improvements in patients with an eating disorder. This pilot study consisted of a very small sample, with 1 patient with anorexia nervosa, and 2 patients with binge eating disorder [25].

In this study we investigated more thoroughly if functionality appreciation is associated with negative body image and another aspect of positive body image, body appreciation, in a sample of (self-reported) eating disorder patients (PAT), recovered eating disorder patients (REC), and controls (HC). Our rationale for including a REC group is to check for symptoms of positive and negative body image disturbance in individuals who have competed their eating disorder treatment. Our previous results suggested that, whilst there is no difference in body attitudes between REC and HC, REC still show perceptual body image disturbances compared to HC [26]. Perceptual body image disturbances, in contrast to negative body attitudes, are not standardly treated in eating disorder treatment but to fully recover from body image disturbances we suggested focussing on perceptual aspects as well. Similarly, functionality appreciation is not (by default) included in standard treatment [24]. If functionality appreciation is indeed positively associated with body appreciation and negatively associated with negative body image, it would suggest that implementing functionality appreciation in standard treatment may be beneficial. To our knowledge, we are the first to have investigated the relationship between positive and negative body image in REC.

In the current study we employed measures of negative body image (appearance dissatisfaction), and measures related to the concept of positive body image (body appreciation and functionality appreciation) [11, 15, 27]. First, we explored between-group differences in positive and negative body image. In line with previous research, we expected PAT to score lowest on measures of positive body image and highest on measures of negative body image compared to HC. We expected REC to show similar levels of negative body image as HC [26]. Since standard treatment for body image predominantly focusses on treating negative body image [23], we expected REC to have similar levels of positive body image as PAT.

Next, we tested the associative relationship between functionality appreciation and appearance satisfaction (negative body image) and body appreciation (positive body image). Given evidence that functionality appreciation improved body image in a non-clinical sample [20], we expected functionality appreciation to be positively associated with positive body image, and negatively associated with negative body image in PAT and REC.

## Method

### Ethics statement

The current study adhered to the tenets of the Declaration of Helsinki [28] and was approved on the 15th of June 2018 by the Faculty Ethics Review Board of Utrecht University, registration number: FETC18-018. Each participant received written information regarding the purpose and procedure of the study. All participants provided signed informed consent before taking part in the study.

### Participants

Participants were recruited through the internet. A link to the questionnaires on the Gorilla.sc [29] platform was posted on the websites of the Leontienhuis, Human Concern, and Stichting JIJ, which are patient organizations and mental health institutions in The Netherlands specialized in eating disorder care, and through proud2bme.nl, a website aimed at providing support for people with an eating disorder and their family. HC were undergraduate students who were recruited through the participant website of the Utrecht University. Undergraduates received course credit for participation.

Groups were formed based on self-report. The PAT group consisted of participants that reported having a current eating disorder and being recently diagnosed with an eating disorder by a psychiatrist, psychologist, or general physician. The REC group consisted of participants that reported having recovered from an eating disorder, had a past eating disorder diagnosis (given by a psychiatrist, psychologist, or general physician), and successfully completed their eating disorder treatment (in other words, no early dropout). Inclusion criteria for HC were no past or present eating disorder diagnosis. Participants were excluded if they did not adhere to the above criteria. Other exclusion criteria were males and age < 18 years.

In total, 1064 participated in the study. For this study, the questionnaire data was used from the 724 participants who completed all the questionnaires. Of this group, 11 people reported not being officially diagnosed by a general physician, psychiatrist, or psychologist, and 2 people reported a disorder that was not an eating disorder. These 13 people did not meet our inclusion criteria and were therefore excluded. A further 14 individuals identified as male and excluded, leading to a total of 697 participants. However, not all participant data was stored correctly due to technical problems with Gorilla.sc [29], leading to missing data for 193 participants. The total sample of this study therefore consisted of 504 participants: 175 HC, 62 patients with anorexia nervosa, 25 patients with other specified feeding or eating disorder, 13 patients with bulimia nervosa, 2 patients with binge eating disorder, 130 recovered anorexia nervosa patients, 59 recovered other specified feeding or eating disorder, 36 recovered bulimia nervosa patients, 2 recovered binge eating disorder patients. Note that diagnostic status was self-reported and was not confirmed in this study. See Table 1 for demographics characteristics of the sample.

**Table 1:** Means, standard deviations, and one-way analyses of variance of demographic and clinical characteristics of the sample

Measure	HC (*n* = 175)	REC (*n* = 102)	PAT (*n* = 227)	*df*	*f*	*p*
	M (SD)	M (SD)	M (SD)			
Age	26.45 (12.32)	28.75 (8.96)	27.21 (9.36)	2, 501	1.57	.210
BMI	22.81 (3.74)	21.85 (5.00)	19.86 (5.25)	2, 476	19.30	< .001
Duration ED	–	7.93 (5.94)	9.50 (9.17)	1, 286	3.45	.064^a^
		*Total (SD)*	*Total (SD)*			
Recovery rate	–	81.61 (14.20)	34.89 (26.00)	1, 315	440	< .001^a^

ED = eating disorder, HC = healthy controls, REC = recovered, PAT = patients. We included anorexia nervosa, other specified feeding or eating disorder, bulimia nervosa and binge eating disorder in our PAT and REC sample

^a^*Welch’s F*

### Questionnaires

#### Demographic and clinical questions

Participants were asked to report gender, age, education, height, and weight. PAT and REC were asked to provide their eating disorder diagnosis that was set by a general physician, psychiatrist, or psychologist. In addition, participants with a current or past eating disorder were asked the duration of their eating disorder in years and their recovery rate, answers could be provided on a slider (VAS scale) ranging from 0 (*eating disorder fully present*) to 100 (*fully recovered*). Note that the numbers were not visible to participants.

#### Multidimensional body-self relations questionnaire-appearance scales

The Multidimensional Body-Self Relations Questionnaire-Appearance Scales (MBSRQ-AS; [30]) was used to assess appearance related aspects of body image (negative body image). The questionnaire consists of 34 items divided over four subscales: Appearance Evaluation, Appearance Orientation, Body area Satisfaction, and Overweight Preoccupation. The items on the first three subscales ranged from 1 (*definitely disagree*) to 5 (*definitely agree*). The items of the Body area Satisfaction subscale ranged from 1 (*very dissatisfied*) to 5 (*very satisfied*). Each subscale was summed.

A high score on Appearance Evaluation indicates positive feelings towards and satisfaction with one’s physical appearance, whereas a low score indicates unhappiness with one’s appearance. A high score on Appearance Orientation indicates that people place more importance on and pay more attention to how they look and engage in extensive grooming behaviours, low scores indicate that individuals pay less attention to how they look. The Overweight Preoccupation subscale measures weight vigilance, fat anxiety, and eating restraint, higher scores are considered more negative. A high score on Body area Satisfaction indicates satisfaction with discrete aspects of one’s appearance and low scores indicate unhappiness with the size or appearance of several body areas. The Cronbach’s coefficient for the MBSRQ-AS ranged from 0.73 to 0.89 in a female sample [30].

#### Body appreciation scale-2

The Body Appreciation Scale-2 (BAS-2; [27, 31]) was used to assess positive body image. The questionnaire consists of 10 items ranging from 1 (*never*) to 5 (*always*). These items were averaged; higher scores reflect a more positive body image. The Cronbach’s coefficient was 0.97 in a sample of women [27].

#### Functionality appreciation scale

Functionality Appreciation Scale (FAS; [21]) was used to assess the appreciation of body functionality. The questionnaire consists of seven items ranging from 1 (*definitely disagree*) to 5 (*definitely agree*). These items were averaged; higher scores reflect a higher appreciation of body functionality. The FAS has a Cronbach’s coefficient of 0.87 [21].

### Data preparation and analysis plan

Negative body image was measured with four subscales of the MBSRQ-AS, each subscale was summed. Internal consistency of each subscale was checked with McDonald’s Omega (ω_t_) (Appearance Evaluation, ω_t_ = 0.93; Appearance Orientation, ω_t_ = 88; Overweight Preoccupation, ω_t_ = 83; Body area Satisfaction, ω_t_ = 0.89). Positive body image was measured with the BAS-2 (ω_t_ = 0.97) and an average score was derived. Functionality appreciation was measured with the FAS (ω_t_ = 0.95), the average score was used.

Data handling and statistical analysis was done in Rstudio [32]. All assumptions were checked before statistical analysis. To compare differences between groups for negative and positive body image, six one-way Anova’s were conducted. *Welch’s F* test was used when the assumption of homogeneity was violated. Post-hoc comparisons were Tukey corrected, or Games-Howell corrected when the assumption of homogeneity was violated [33].

To assess whether FAS was associated with positive body image (BAS-2) and negative body image (the 4 MBSRQ-AS subscales), we first centered the FAS mean scores with the r base scale function. We then derived Type II analysis of deviance tables using the Anova function from the car package [34]. To further explore the significant interactions, 4 simple slope analyses were conducted with the emtrends function from the emmeans package [35].

## Results

### Demographic and clinical characteristics

An ANOVA revealed significant between group differences for BMI for HC, REC and PAT, see Table 1 for statistics. Post-hoc comparisons revealed that the HC group had a significant higher BMI, compared to PAT (*p* < 0.001), but not to REC (*p* = 0.25). REC had a significant higher BMI compared to PAT (*p* = 0.002). A *Welch’s F* test revealed significant differences in recovery rate between groups, see Table 1 for statistics. Post-hoc comparisons revealed that REC rated themselves as significantly more recovered than PAT.

### Body image between groups

**Fig. 1:**
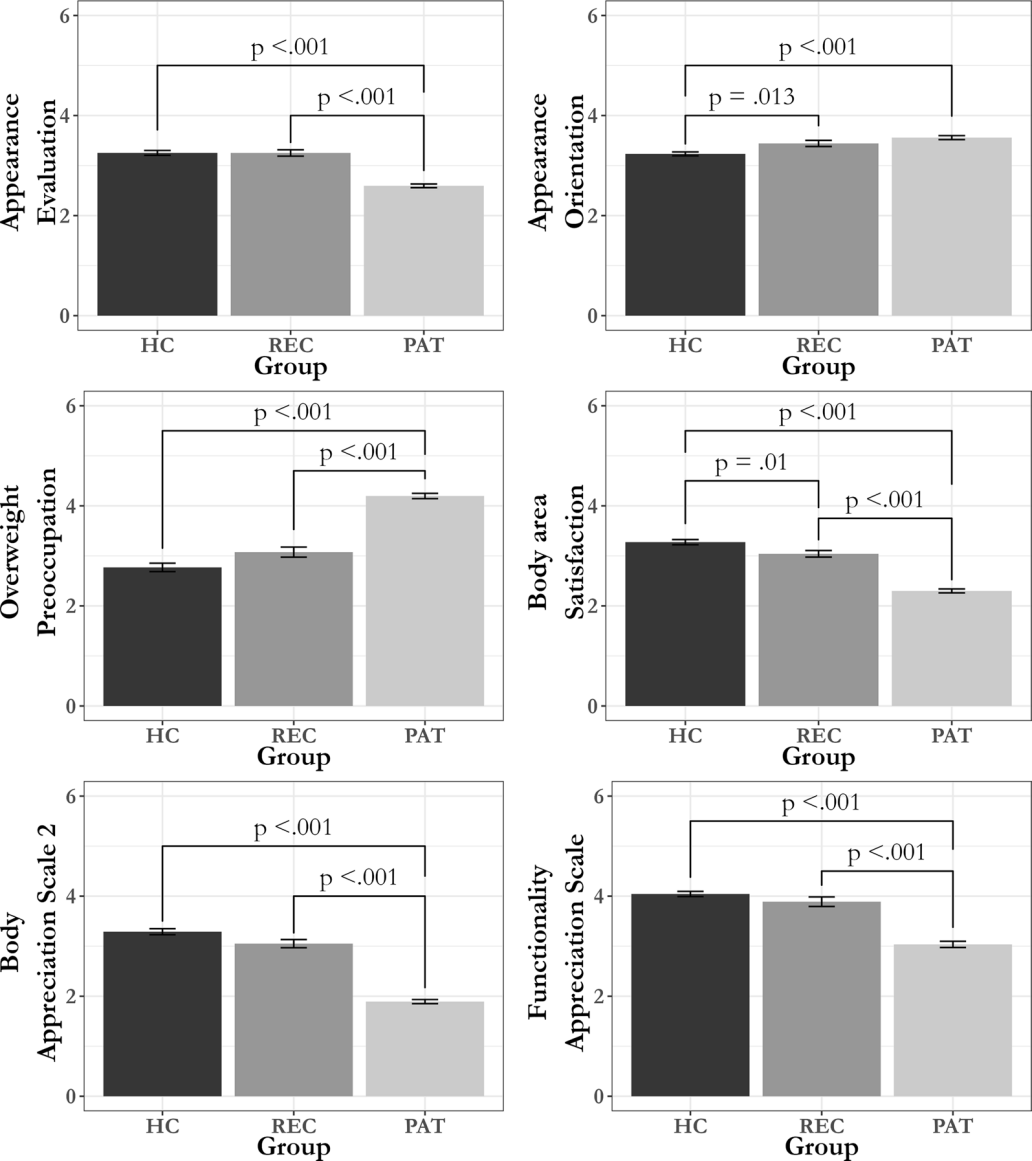
Post hoc comparisons with Tukey correction of the Multidimensional Body-Self Relations Questionnaire-Appearance Scales [36], Body Appreciation Scale-2 [27, 31]) and Functionality Appreciation Scale [21]. Error bars represent SE. HC = healthy controls, REC = recovered, PAT = patients

Differences in mean scores between groups were found for all subscales of the MBSRQ-AS and BAS-2 and FAS, see Table 2 for means and statistics. Post-hoc comparisons revealed significant differences between PAT and HC for all outcome variables. HC had a significantly lower score for Appearance Orientation compared to REC and PAT, no significant differences were found between REC and PAT for this subscale. HC had higher scores for the Body area Satisfaction scale compared to REC and REC scored higher compared to PAT. Significant differences between REC and PAT were found for Appearance Evaluation, Overweight Preoccupation, BAS-2, FAS, where PAT scored more negatively compared to REC. No significant differences were found for these subscales between REC and HC. See Fig. 1 for post-hoc comparisons.

**Table 2:** Means, standard deviations, and one-way analyses of variance of the Multidimensional Body-Self Relations Questionnaire-Appearance Scales [36], Body Appreciation Scale-2 [27, 31]) and Functionality Appreciation Scale [21]

Measure	HC (*n* = 175)	REC (*n* = 102)	PAT (*n* = 227)	*df*	*f*	*p*
	*M (SD)*	*M (SD)*	*M (SD)*			
AE	3.25 (0.65)	3.25 (0.64)	2.60 (0.55)	2, 252	76.00	< .001^a^
AO	3.23 (0.52)	3.44 (0.62)	3.56 (0.60)	2, 259	17.20	< .001^a^
OP	2.77 (1.12)	3.08 (1.02)	4.20 (0.79)	2, 243	124.00	< .001^a^
BS	3.27 (0.69)	3.04 (0.67)	2.30 (0.60)	2, 501	123.30	< .001
BAS-2	3.29 (0.80)	3.05 (0.83)	1. 89 (0.61)	2, 241	215.00	< .001^a^
FAS	4.04 (0.69)	3.89 (0.96)	3.04 (0.94)	2, 255	80.20	< .001^a^

HC = healthy controls, REC = recovered, PAT = patients, AE = Appearance Evaluation, AO = Appearance Orientation, OP = Overweight Preoccupation, BS = Body area Satisfaction, BAS-2 = Body Appreciation Scale-2, FAS = Functionality Appreciation Scale

^a^*Welch’s F*

Overall, PAT scored more negatively on both positive and negative body image measures and on functionality appreciation compared to HC. There were no differences in scores between REC and HC in positive and negative body image measures and the FAS, with an exception for the subscale Appearance Orientation and Body area Satisfaction. These scores indicate that REC were more oriented towards their appearance and less satisfied with specific body areas compared to HC.

### Functionality appreciation and body image

There were significant interactions between FAS and Group for Appearance Evaluation, Overweight Preoccupation, Body area Satisfaction and the BAS-2. There was no significant interaction between FAS and Group for Appearance Orientation. Furthermore, main effects were found for both FAS and Group for all outcome variables. See Table 3 for inferential statistics.

**Table 3:** Results of the ANOVAS

Variable	*df*	*f*	*p*	*η*_*p*_^*2*^
*Appearance Evaluation*				
FAS	1, 498	42.464	< .001	.08
Group	2, 498	33.425	< .001	.12
FAS:Group	2, 498	13.850	< .001	.05
*Appearance Orientation*				
FAS	1, 498	10.130	.002	.02
Group	2, 498	7.192	< .001	.03
FAS:Group	2, 498	0.186	.830	.00
*Overweight Preoccupation*				
FAS	1, 498	49.64	< .001	.09
Group	2, 498	59.44	< .001	.19
FAS:Group	2, 498	4.95	.007	.02
*Body area Satisfaction*				
FAS	1, 498	148.578	< .001	.23
Group	2, 498	48.539	< .001	.16
FAS:Group	2, 498	3.534	.030	.01
*Body Appreciation Scale-2*				
FAS	1, 498	309.808	< .001	.38
Group	2, 498	101.327	< .001	.29
FAS:Group	2, 498	10.121	< .001	.04

FAS = Functionality Appreciation Scale [21]

**Fig. 2:**
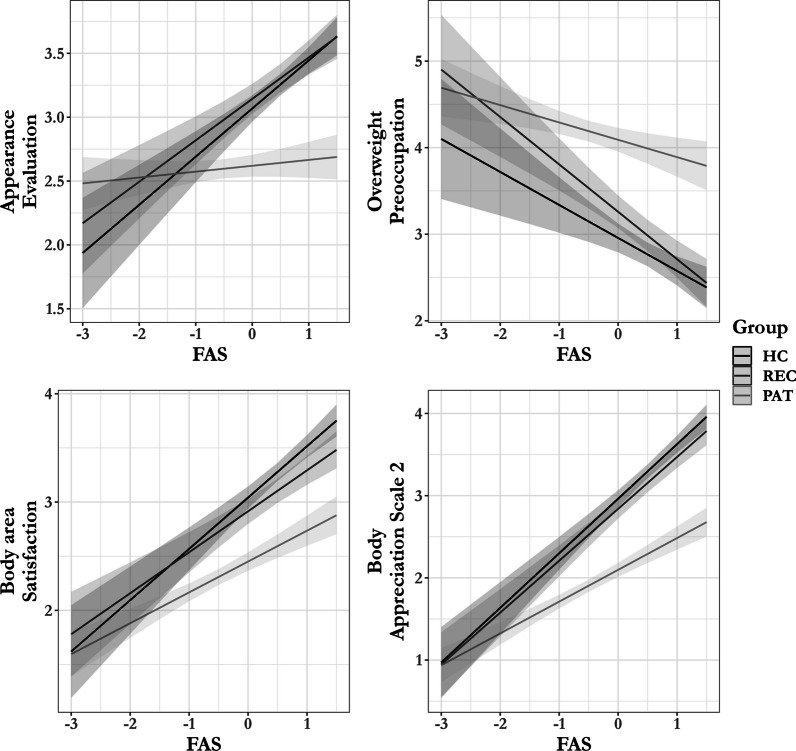
Regression slopes of the estimated marginal means of the Multidimensional Body-Self Relations Questionnaire-Appearance Scales [36], Body Appreciation Scale-2 [27, 31] and Functionality Appreciation Scale [21]. FAS = Functionality Appreciation Scale, HC = healthy controls, REC = recovered, PAT = patients. FAS mean scores were centered

To further examine the significant interactions, we conducted simple slope analysis. Simple slope analysis showed that the FAS is significantly associated with Appearance Evaluation, Overweight Preoccupation, Body area Satisfaction and the BAS-2, for HC and REC. For PAT the FAS was also found to be significantly associated with Overweight Preoccupation, Body area Satisfaction and the BAS-2, but not Appearance Evaluation. See Fig. 2 and Table 4 for statistics.

**Table 4:** Inferential statistics of the Simple Slopes Analysis

Group	*FAS.trend*	*SE*	*Df*	*t-ratio*	*p*	*CI*
*Appearance Evaluation*						
HC	0.377	0.062	498	6.107	< .001	[0.256, 0.498]
REC	0.324	0.058	498	5.619	< .001	[0.214, 0.438]
PAT	0.046	0.040	498	1.157	.248	[-0.032, 0.124]
*Overweight Preoccupation*						
HC	−0.381	0.099	498	−3.842	< .001	[−0.576, −0.186]
REC	−0.548	0.093	498	−5.861	< .001	[−0.730, −0.365]
PAT	−0.200	0.064	498	−3.186	.002	[−0.326, −0.075]
Body area Satisfaction						
HC	0.474	0.061	498	7.736	< .001	[0.353, 0.594]
REC	0.381	0.057	498	6.602	< .001	[0.266, 0.491]
PAT	0.282	0.039	498	7.226	< .001	[0.207, 0.362]
Body Appreciation Scale-2						
HC	0.664	0.062	498	10.756	< .001	[0.543, 0.785]
REC	0.631	0.058	498	10.933	< .001	[0.518, 0.745]
PAT	0.387	0.040	498	9.738	< .001	[0.309, 0.465]

In line with expectations these results indicate that the FAS is significantly associated with positive and negative body image across groups. In addition, we did not find that the FAS was significantly associated with appearance evaluation in PAT, whereas it was a significant predictor amongst REC and HC.

## Discussion

The current study investigated positive and negative body image in a sample of PAT, REC and HC. In line with previous literature, we measured negative body image in terms of appearance dissatisfaction (specifically: appearance evaluation, appearance orientation, overweight preoccupation, and body area satisfaction), and measured two aspects of positive body image (body appreciation and functionality appreciation) [11, 15, 22].

In line with expectations and previous literature, results revealed that PAT have a more negative and a less positive body image compared HC [13, 14]. Results further showed that REC do not differ from HC in terms of functionality appreciation, body appreciation and most measures of negative body image, except appearance orientation and body satisfaction. REC were significantly more appearance oriented compared to HC. For body satisfaction, results showed that REC were significantly more satisfied with discrete aspects of their appearance compared to PAT but not to the same extent as HC.

In contrast to expectations, results showed no differences between PAT and REC for appearance orientation (which measures the importance one places on their appearance). These results indicate that REC place the same importance on their appearance as PAT. At first glance this seems surprising as treatment for eating disorders is often directed at reducing over-evaluation of shape and weight (e.g., CBT-E) [12]. One explanation for this elevated score in REC could be that REC might indeed place the same amount of importance on their appearance but, at the same time, are more satisfied with their appearance, appreciate their body, and appreciate its functions more than PAT. In other words, REC might still place importance on their appearance and are less satisfied with specific body parts, but this might not be necessarily problematic as they are not more dissatisfied with their overall appearance than HC. This possibility is consistent with findings indicating that of all appearance related concepts, dissatisfaction with one’s appearance is the most prominent predictor of eating pathology and relapse [4–6, 37]. However, some clinicians claim that a roadblock to successful treatment is the way in which appearance (specifically, thinness) becomes tied to their patient’s identities, such that it is viewed as of paramount importance [38]. Given that our recovered group did not appear to have overcome this feature of the disorder, our results suggest either that it is not, ultimately, most important for achieving recovery or puts into question the recovered status of those who have successfully completed treatment (such as those in our recovered group).

In addition to group differences, we also tested for a relationship between functionality appreciation and body appreciation (positive body image), and an inverse relationship between functionality appreciation and appearance satisfaction (negative body image). In line with our expectations, results revealed that functionality appreciation was positively associated with body appreciation. This is not surprising given that the two constructs overlap by definition [22]. We further found that functionality appreciation was negatively associated with of most measures of negative body image. However, the strength of this relationship depended on group membership for measures of appearance evaluation, overweight preoccupation, satisfaction with body areas (aspects of negative body image), and body appreciation.

Group membership did not significantly influence the strength of the relationship between functionality appreciation and appearance orientation (an aspect of negative body image). Specifically, our results revealed that functionality appreciation was negatively associated with overweight preoccupation, while positively associated with satisfaction with body areas, and body appreciation for PAT, REC and HC. Interestingly, functionality appreciation was also found to be positively associated with appearance evaluation for HC and REC, but not PAT. In other words, functionality appreciation does not appear to be associated with PATs’ feelings of satisfaction or dissatisfaction with their appearance. The absence of an effect in PAT is interesting as previous research showed reductions in body dissatisfaction, in both a non-clinical and clinical sample, after taking part in intervention focusing on body functionality [20, 25].

One possibility for why we did not find a relation between functionality appreciation and appearance evaluation in our patient group is that we had a one-time measure of these variables. This relationship might need to be developed through practice. For example, in a previous study Alleva et al., [20, 39], found evidence to suggest that functionality appreciation only increases appearance satisfaction after extended training (consisting of a three-session writing task). In a clinical sample, effects were found using an intervention that lasted several weeks (tailored to individual needs) with 4 sessions a week [25]. In other words, while the relationship between functionality appreciation and appearance evaluation might not be present amongst PAT in our sample, it may still be possible to establish through practice.

We did find a relationship between functionality appreciation and appearance *evaluation* in REC. Unfortunately, we do not know if REC in our sample participated in a functionality appreciation training. Even though functionality appreciation is not usually included in body image treatment for eating disorders [24], we cannot rule out that it featured in the treatment that our sample underwent. Consequently, further research needs to be done to investigate the (possible) relationship between body functionality and appearance satisfaction in patients and REC.

Apart from appearance evaluation, we did find several significant relationships between functionality appreciation and body appreciation and aspects of negative body image in our sample. This is an encouraging finding as it indicates that increasing functionality appreciation might be used as an intervention to increase positive and decrease negative body image in PAT. Consistent with this, a recent pilot study using such interventions in a clinical sample has shown promising results [25]. However, it should be noted that our research is based on observational data and it is therefore difficult to draw conclusions regarding causal effects. Further experimental research should investigate the beneficial effects of functionality appreciation interventions in large-scale clinical settings.

Our study was not without limitations. One limitation of this study is that we formed groups based on self-reported diagnosis, that were not confirmed. Even though our demographic information matched with our groups – our REC group also reported higher levels of recovery compared to PAT (see Table 1) – we did not verify this with a diagnostic tool. However, we recruited our participants from websites from eating disorder clinics that are frequently visited by patients. Such recruitment methods have been used in other studies, which found that self-reported eating disorder matched the outcome of their diagnostic tool (only 7 participants were excluded of 318 because they did not meet the diagnostic requirements) [40].

Another limitation is that this study did not measure REC prior to treatment, therefore, we cannot infer a causal link between body image and eating disorder treatment. However, based on the patient sample, we speculate that both negative and positive body image, except for appearance orientation, improved after eating disorder treatment.

## Conclusions

We did not find any differences in body image (positive and negative) in REC compared to HC, except for appearance orientation and body area satisfaction (negative body image). Here we found that both REC and PAT valued their appearance significantly more than HC. For body area satisfaction, we found that HC were more satisfied with body areas compared to REC. In addition, we found that functionality appreciation was positively associated with positive body image and negatively associated with negative body image, except for appearance evaluation, in PAT. This study confirms previous research associating functionality appreciation with improvements in body image. Functionality appreciation might be a useful tool in optimizing treatment for body image. However, research needs to be conducted to investigate the effectiveness of functionality appreciation interventions in large-scale clinical settings for eating disorders.

## Declarations

### Availability of data and materials

Data is available on request from the authors.
